# Bovine CCL28 Mediates Chemotaxis via CCR10 and Demonstrates Direct Antimicrobial Activity against Mastitis Causing Bacteria

**DOI:** 10.1371/journal.pone.0138084

**Published:** 2015-09-11

**Authors:** Kyler B. Pallister, Sara Mason, Tyler K. Nygaard, Bin Liu, Shannon Griffith, Jennifer Jones, Susanne Linderman, Melissa Hughes, David Erickson, Jovanka M. Voyich, Mary F. Davis, Eric Wilson

**Affiliations:** 1 Department of Immunology and Infectious Diseases, Montana State University, Bozeman, Montana, United States of America; 2 Department of Microbiology and Molecular Biology, Brigham Young University, Provo, Utah, United States of America; INRA, UR1282, FRANCE

## Abstract

In addition to the well characterized function of chemokines in mediating the homing and accumulation of leukocytes to tissues, some chemokines also exhibit potent antimicrobial activity. Little is known of the potential role of chemokines in bovine mammary gland health and disease. The chemokine CCL28 has previously been shown to play a key role in the homing and accumulation of IgA antibody secreting cells to the lactating murine mammary gland. CCL28 has also been shown to act as an antimicrobial peptide with activity demonstrated against a wide range of pathogens including bacteria, fungi and protozoans. Here we describe the cloning and function of bovine CCL28 and document the concentration of this chemokine in bovine milk. Bovine CCL28 was shown to mediate cellular chemotaxis via the CCR10 chemokine receptor and exhibited antimicrobial activity against a variety of bovine mastitis causing organisms. The concentration of bovine CCL28 in milk was found to be highly correlated with the lactation cycle. Highest concentrations of CCL28 were observed soon after parturition, with levels decreasing over time. These results suggest a potential role for CCL28 in the prevention/resolution of bovine mastitis.

## Introduction

Effective immune surveillance and protection is reliant on the efficient homing, accumulation and positioning of immune cells. The homing of immune cells is mediated through a multi-step process involving the vascular expression of adhesion molecules and chemokines, as well as leukocyte expression of cognate adhesion molecule ligands and chemokine receptors [1]. Chemokines, as their name implies, are chemotactic for cells which express the appropriate receptors [2]. The chemokine CCL28, also known as mucosal epithelial chemokine (MEC), binds the CCR3 and CCR10 chemokine receptors [3,4]. CCR10/CCL28 interactions have been shown to be essential for efficient accumulation of antigen specific IgA plasma cells to the murine large intestine and mammary gland [5–8].

In addition to the well-established role of chemokines in leukocyte homing and migration, several chemokines have been shown to exhibit antimicrobial properties. These chemokines include: CCL20, CXCL9, CXCL10, CXCL11, CCL6 and CCL28 [9–12]. The chemokine CCL28 has been shown to exhibit potent antimicrobial activity against both Gram-positive and Gram-negative bacterial pathogens *in vitro* [11,13]. Many antimicrobial peptides (AMPs), including antimicrobial chemokines, are positively charged. It has been hypothesized that recognition of bacterial targets by AMPs is mediated through electrostatic interactions of the positively charged AMP with negatively charged molecules on the bacterial membrane [14]. Consistent with this hypothesis, previous research has demonstrated that the C-terminal end of CCL28 is positively charged and a specific sequence (RKDRK) is essential to the antimicrobial function of murine CCL28 (mCCL28) [13].

We have previously demonstrated that bovine CCL28 (bCCL28) mRNA is expressed in mucosal tissues including the mammary gland [15]. The mucosal expression patterns observed for bCCL28 suggest that it likely serves a similar function in the cow as CCL28 does in other better characterized animal models [4,6,7,11,16–20]. However, data describing the function and possible role of bCCL28 has not been previously published.

Mastitis, caused by infection of the lactating mammary gland, is the most costly production disease of dairy cattle [21]. In an effort to better understand the potential role of CCL28 in preventing/combating bovine mastitis, we cloned and expressed bCCL28 and tested the function of this protein in both chemotaxis and antimicrobial assays. Results demonstrate that bCCL28 possesses chemotactic activity, mediating the migration of CCR10 receptor bearing cells. These data suggest that bCCL28 may play a key role in the migration of antibody secreting cells to bovine mucosal tissues, including the mammary gland. Furthermore, we show that bCCL28 has potent *in vitro* antimicrobial activity against microorganisms known to cause mastitis in dairy cattle, including *Staphylococcus aureus*, *Streptococcus uberis*, *Streptococcus agalactiae*, *Escherichia coli*, and *Pseudomonas aeruginosa*. The observed chemotactic and antimicrobial properties of bCCL28 indicate that this chemokine may contribute to immune protection of the mammary gland, and the resolution or prevention of bovine mastitis.

## Methods

### Recombinant Chemokines

All animal tissues were acquired from a slaughterhouse; no animals were euthanized for the purpose of obtaining tissues for this study. To carry out these studies, molecular constructs of bovine chemokines were generated. For chemotaxis experiments, full-length bCCL28 cDNA was cloned from mRNA collected from bovine parotid salivary gland. Bovine CCL27 (bCCL27), used in some experiments, was cloned from bovine skin. Sequences for all primers are contained in Table 1. CCL28 cDNA was then cloned into the pVAX-1 vector (Life Technologies, Grand Island, NY, USA). All sequences were confirmed through cycle sequencing and comparison to the NCBI database (accession number EF654535 for bCCL28 and accession number XM_585789 for bCCL27). Recombinant protein was produced through transfection of Cos-7 cells to ensure proper folding of the protein. Cos-7 cells were transfected with mCCL28-pVAX, bCCL28-pVAX, or empty pVAX vector using Lipofectamine 2000 (Life Technologies, Carlsbad, CA, USA). Transiently transfected Cos-7 cells were then cultured in Roswell Park Memorial Institute medium (RPMI) supplemented with 10% fetal bovine serum (Life Technologies) for 48hr. Supernatants from transfected cell cultures were then concentrated using Amicon centrifugal filter devices (Millipore, Billerica, MA, USA) with a 3kD molecular weight filter. Concentrated chemokine and control supernatants were then used in migration assays as described below.

**Table 1:** Primer names and sequences.

Primer Name	Sequence (5’-3’)
pVAX bCCL27F	GACGGAATTCGCCGCCACCATGTCGGGATTGAGGAGATACGAG
pVAX bCCL27R	CAGGGAATTCTTACTTTGGCTTCTGGGGGCCCCAG
pVAX bCCL28F	GACGGAATTGCGGGCCACCATGGGAATGCAGCAGACAGGACTC
pVAX bCCL28R	AGGGAATTCCTAATAAGGCGTTTTGTGGCCATGTG
pET19b bCCL27F	GACGCTCGAGTACCGACAGCCACTCCCAAACAAGC
pET19b bCCL27R	GGACCTCGAGTTACTTTGGCTTCTGGGGGCCCCAG
pET19b bCCL28F	GACGCTCGAGATACTTCCCATTGCCTCCAGCTGCTG
pET19b bCCL28R	CAGGCTCGAGCTAATAAGGCGTTTTGTGGCCATGTGTTTC

For antimicrobial assays, a second construct of bCCL28 was made which included a His tag to facilitate purification and quantification of the protein. This construct excluded the signal peptide. As a control, the closely related chemokine bCCL27 was cloned and expressed. Proteins were expressed in *E*. *coli* as N-terminal His-tagged fusion proteins through cloning into the XhoI site of the pET19b expression vector (Novagen, Inc., Madison, WI, USA) as previously described [13]. Briefly, the chemokine-coding cDNA sequence without its signal sequence was amplified by PCR, cloned into the XhoI site of pET19b, and the resulting plasmids were confirmed through cycle sequencing. All engineered pET19b plasmids were transformed into *E*. *coli* BL21 (DE3) cells for protein production. Recombinant protein was harvested from 1 L cultures of bacteria grown for 12–18hr in Luria Broth supplemented with Isopropyl β-D-1-thiogalactopyranoside (IPTG) (1 mM). Bacteria were harvested by centrifugation at 4000 x g (4°C) for 10 min and pellets were resuspended in 60 mL of 0.3 M NaCl/10 mM Imidazol/20 mM Tris, pH8. In order to purify recombinant bCCL28 from inclusion bodies, bacteria were lysed by sonication on ice for 15 minutes at 30% amplitude with pulsing at 1-second intervals. Samples were centrifuged at 10,000 x g for 10 minutes, supernatants discarded, and pelleted cell debris washed with dH_2_O, followed by resuspension in 60 mL 7.5 M Urea/0.5 M NaCl in 20 mM Tris, pH8. The sonication and centrifugation steps were repeated and supernatant was loaded onto a nickel-nitrilotriacetic acid column. Recombinant protein was purified according to the manufacturer’s protocol (His SpinTrap^TM^, GE Healthcare, Buckinghamshire, UK). The purified His-tag fusion protein solution was then dialyzed against 20 mM Tris-HCL buffer (pH8) overnight in a 5 L beaker with three buffer changes. The dialyzed protein solution was loaded onto a S.P. Sepharose Fast Flow column (GE Healthcare) and purified further as per manufacturers protocol. Eluted fractions containing bCCL28 or bCCL27 were dialyzed as above and concentrated using Centrifugal Filter Units (Millipore, Billerica, MA, USA). The purity of the protein was confirmed by visualizing the protein by Coomassie staining following electrophoresis on a 12% SDS-PAGE gel. Staining revealed a single band of protein at the expected molecular weight of bovine CCL28 (~13 kD). The concentration of the protein was determined by Bradford assay (Thermo, Sci., Rockford, IL, USA).

### Migration Assay

Supernatant fluids from Cos-7 cells, transiently transfected with empty vector or the mCCL28 or bCCL28 expression plasmids, were collected 48hr post transfection. Supernatant fluids were then used to test the ability of recombinant bovine chemokines to induce migration of CCR10 expressing cells. In these assays L1.2 cells transfected with mCCR10 were employed. Briefly, 600μl of concentrated supernatant fluids were placed in the bottom well of a Transwell migration chamber (Costar, Corning, NY, USA). Chemokine receptor transfected L1.2 cells (100,000 in a volume of 100 μL) were then placed in the upper chamber of the Transwell and cells were allowed to migrate for 1.5hr at 37°C. Following incubation, migrated cells were harvested from the lower chamber and the percent of migratory cells was determined by flow cytometry, as previously described [7,20].

### Antimicrobial Peptide Assays

Five different species of bacteria were used in this study: Methicillin-resistant *Staphylococcus aureus* (pulsed-field gel electrophoresis type USA 300, strain LAC); *Streptococcus uberis* (*S* ATCC #BAA-854); *Streptococcus agalactiae* (ATCC # 7077); *Pseudomonas aeruginosa* (QMP W1-212); and *Escherichia coli* (DH5α). The isolates of *S*. *uberis*, *S*. *agalactiae* and *P*. *aeruginosa* used in this study were all originally isolated from mastitic cattle. *Staphylococcus aureus* bacteria were grown at 37°C in tryptic soy broth (TSB); Streptococcal species were grown in Todd Hewitt Broth and *E*. *coli* were grown in Luria Broth. All bacteria were grown to mid-log phase (OD_600_). Bacterial cells were then resuspended at ~10^5^ colony forming units/mL (CFUs/mL) in 1 mM Tris pH8 containing 1.0% TSB and incubated for one hour at 37°C with an equal volume of buffer with or without varied doses of recombinant chemokines. Percent survival was determined by plating bacteria on tryptic soy agar (for *S*. *aureus* and *P*. *aeruginosa*), brain heart infusion agar (for *Streptococcus* species) or Luria agar (for *E*. *coli*). CFUs were enumerated the following day and the percentage of bacteria that survived was calculated using the equation: (CFU_pathogen + chemokine_/CFU_pathogen only_) x 100. All data were obtained by performing three or more separate experiments

### ELISA Assay

CCL28 levels were determined by ELISA. Briefly, milk samples were obtained from a local dairy farm and diluted 1:10 in PBS. All reagents used in ELISA assays were marketed as the Human CCL28 DuoSet ELISA kit, although designed for detection of human CCL28, these reagents were found to cross react with bCCL28 with a sensitivity of ~5ng/ml (R&D Systems, Minneapolis, MN, USA). ELISA plates were coated with 100μL mouse anti-human CCL28 antibody diluted in PBS and incubated overnight at room temperature (RT). The plates were then washed three times with wash buffer (0.05% Tween 20 in PBS) and then blocked with blocking buffer (1% BSA in PBS) for 3hr. The plates were washed three times and then incubated overnight with 100μL diluted milk sample or known concentrations of recombinant bCCL28. After three washes, wells were incubated with 100μL biotinylated goat anti-human CCL28 antibody (R&D Systems) diluted in blocking buffer for 2 hours RT. Wells were subsequently washed three times. Wells were then incubated with streptavidin-conjugated horse radish peroxidase (HRP) (BD Biosciences, San Jose, CA, USA) diluted in blocking buffer for 20 minutes at RT. Wells were again washed three times and incubated with TMB Substrate (BD Biosciences) for 20 to 40 minutes. Reactions were then stopped with 50μL 2N H2SO4 and the OD_450_ measured. CCL28 concentrations were determined using a standard curve generated from known values.

### Milk Samples

CCL28 levels were determined on individual milk samples from 80 Holstein or Holstein cross cows during various points in the lactation cycle. All milk samples were collected during routine milking by dairy employees (no samples were collected specifically for these experiments). In addition to milk samples, information on somatic cell count, days post calving, and number of lactations was provided by the dairy (Prior Dairy, Springville, Utah, USA). Data on somatic cell counts were generated as part of an ongoing dairy herd monitoring program conducted by the dairy owner in conjunction with the National Dairy Herd Information Association (Wellsville, UT, USA).

### Statistical Analysis

Statistical analyses for migration and antimicrobial assays were performed using Graphpad Prism (version 5 Graphpad software) using a Mann-Whitney *U* test. Linear regression analysis was performed using Excel. A value of *p <*0.05 was considered significant in all analyses.

## Results

### Bovine and murine CCL28 exhibit minimal homology at the antimicrobial C-terminus

**Fig 1:**
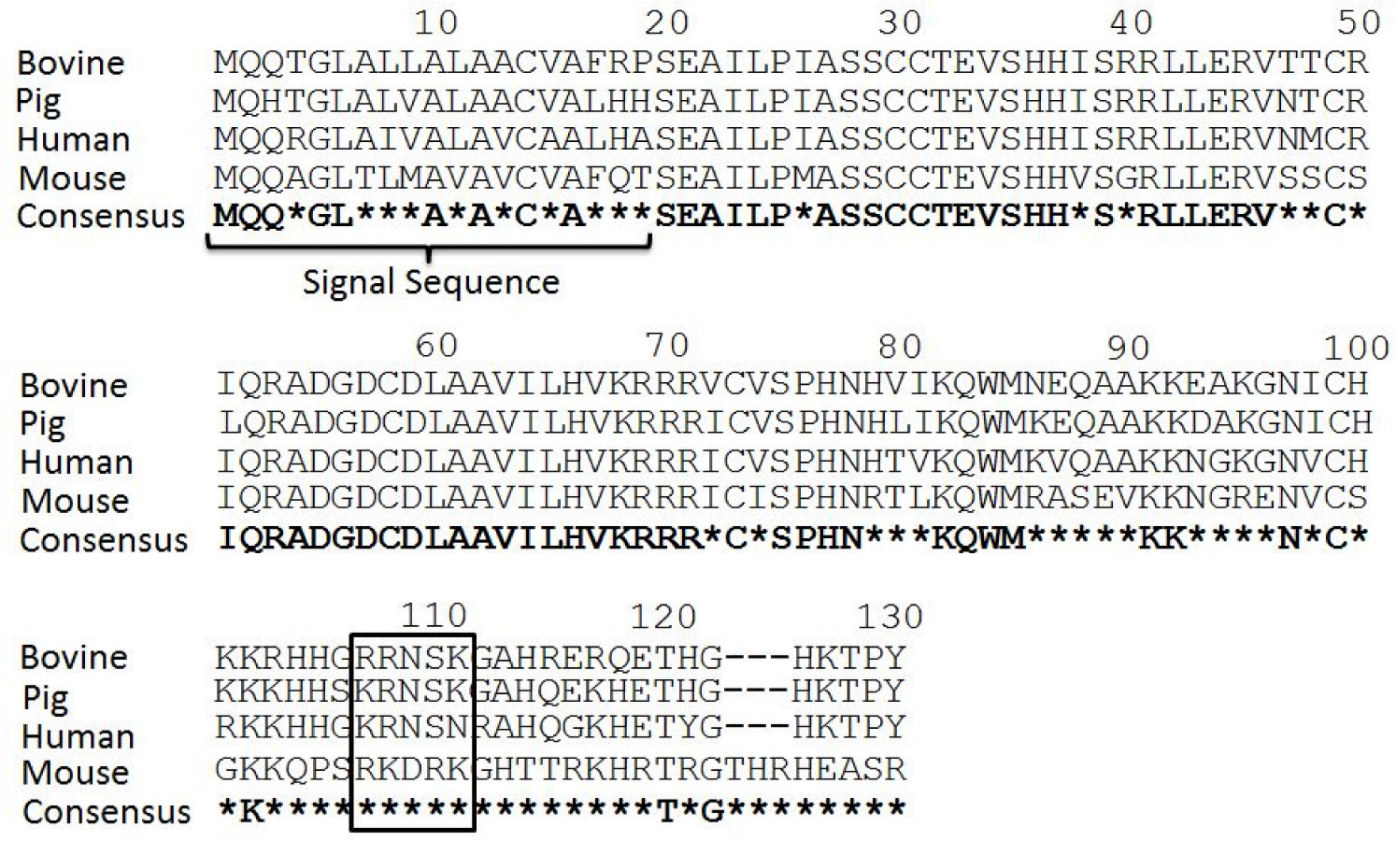
Alignment of bovine and murine CCL28. Alignment of bovine, pig, murine and human CCL28 sequences demonstrates high amino acid homology at the N-terminus of the protein and lower homology at the C-terminus. Asterisks (*) denote amino acid changes between murine, pig, human and bovine CCL28 sequences. The RKDRK sequence, which has previously been shown to be essential for optimal antimicrobial function of mCCL28 and corresponding bovine sequence, is boxed.

Chemokines are essential in the migration of lymphocytes. Additionally, some chemokines have been shown to act as antimicrobial peptides. It has been hypothesized that the N-terminal region of CCL28 and other chemokines mediates chemotactic activity and the C-terminal region mediates the antimicrobial activity of the protein [13,22–24]. The antimicrobial activity of mCCL28 has been shown to be primarily dependent on a five amino acid sequence (RKDRK) [13]. Alignment of human, mouse, pig and bovine CCL28 demonstrates strong homology at the N-terminal end of the protein and less homology at the C-terminal end. The RKDRK region of the mouse protein, which has been shown to be essential for AMP function, is altered to RRNSK in the bovine protein (Fig 1).

### Bovine CCL28 mediates migration via the CCR10 chemokine receptor

**Fig 2:**
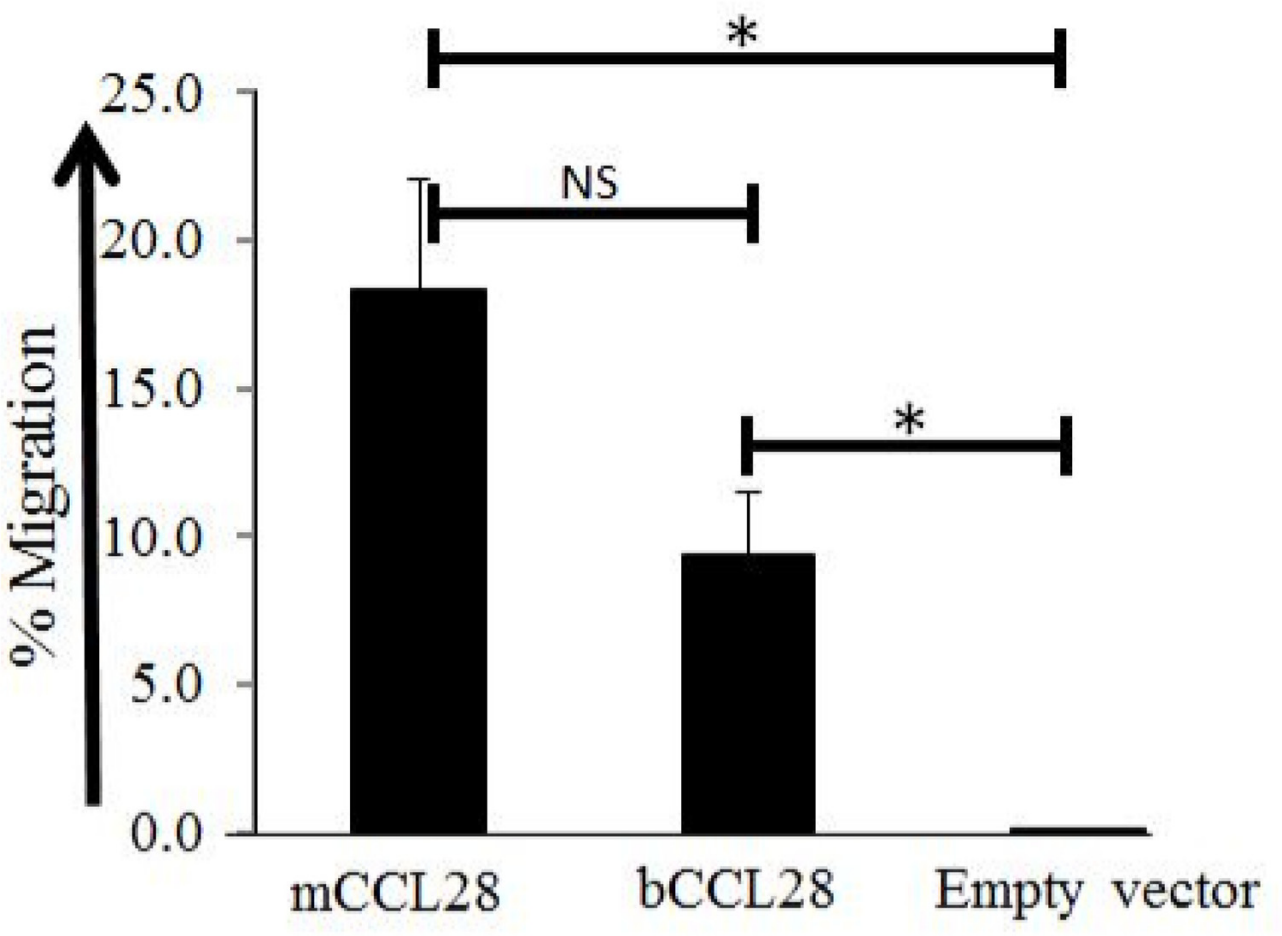
Bovine CCL28 mediates migration of CCR10 transfected cells Supernatant fluids from Cos-7 cells transfected with mCCL28, bCCL28, or empty vector controls were placed in the bottom well of a Transwell migration chamber. Cells expressing mCCR10 were placed in the upper chamber and allowed to migrate for 1.5hrs. Both murine and bovine CCL28 mediated migration of CCR10 transfectants significantly better than empty vector controls *p<0.05, as determined by Mann Whitney *U* test. Results are from four separate experiments. Average migration of cells migrating to mCCL28 was 18.4% (SE 3.71), migration to bCCL28 9.4% (SE 2.12), and migration to empty vector 0.1% (SE 0.01). Error bars represent standard error of the mean. NS = Not Significant.

Mouse CCL28 has been shown to play an essential role in the migration and accumulation of plasma cells into mucosal tissues [7,11,20,25]. To address the potential role of bCCL28 in mediating the migration of lymphocytes via the CCR10 chemokine receptor, recombinant protein was used in Transwell chemotaxis assays. In these assays bCCL28 efficiently mediated lymphocyte chemotaxis via the CCR10 chemokine receptor (Fig 2). These data establish the ability of bCCL28 to mediate the migration of CCR10 receptor expressing lymphocytes.

### Bovine CCL28 exhibits antimicrobial activity

**Fig 3:**
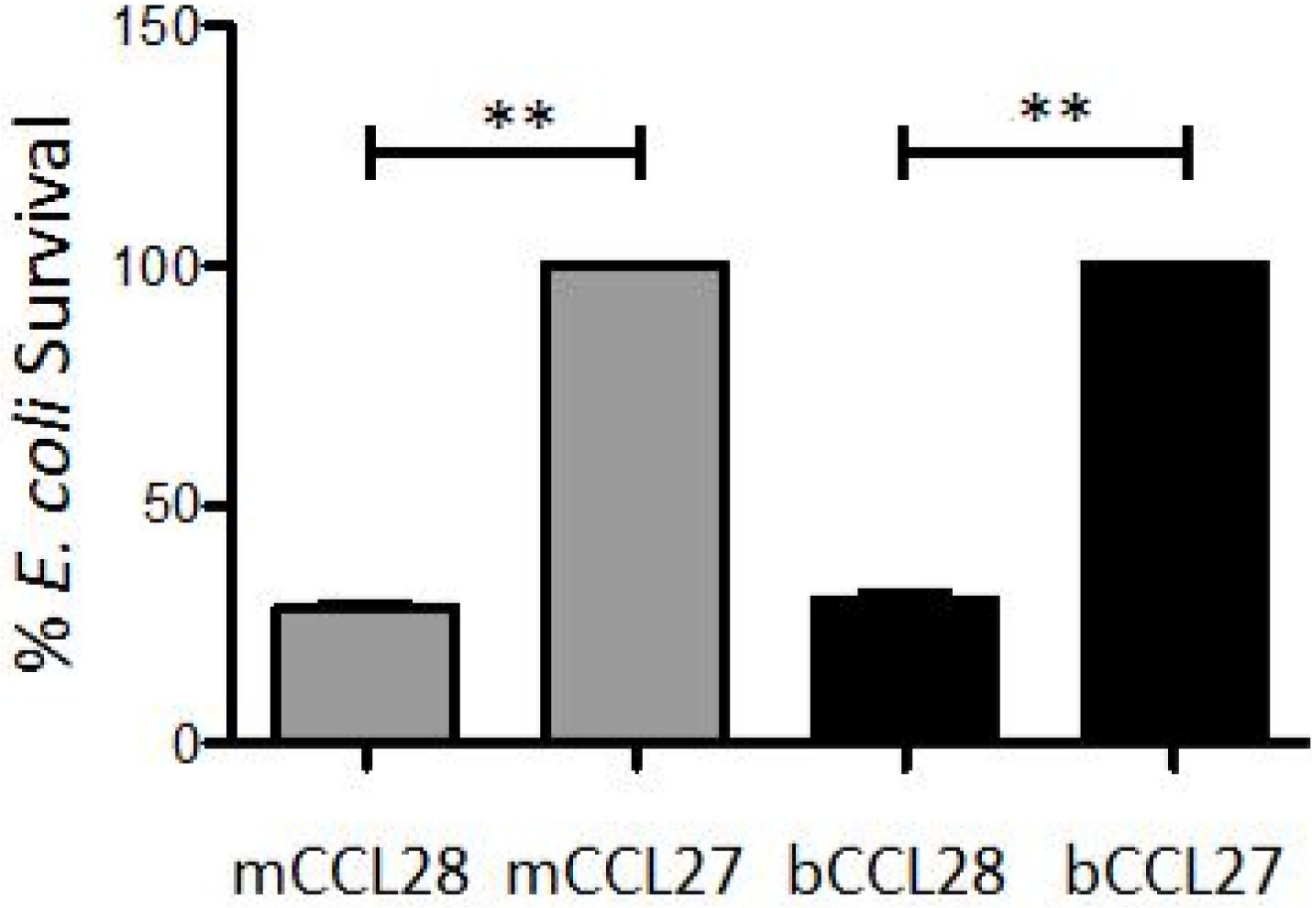
Recombinant bCCL28 exhibits antimicrobial activity *in vitro*. *E*. *coli* was incubated with 0.25 μM of mouse or bovine CCL28 or CCL27 for 2 hours. Percent survival was calculated using the following equation: (CFU_pathogen + chemokine_/CFU_pathogen only_) x 100. **p<0.01 as determined by Mann Whitney *U* test. Results are from three separate experiments, each performed in duplicate. Survival of bacteria in the CCL27 treated groups was scaled to 100% and the amount of killing in the CCL28 treated groups is shown as percent survival compared to the CCL27 treated group. Average bacterial survival seen in mCCL28 treated bacteria 28.3% (SE 1.35), survival observed in bCCL28 treated bacteria 30.3% (SE 1.29). Error bars represent standard error of the mean.

The amino acid sequence of bCCL28 has strong homology to mouse and human CCL28 in the N-terminal region with the bovine protein exhibiting 93% identity to the human protein between the end of the signal sequence to amino acid 80 (Fig 1) [13]. However, within the antimicrobial C-terminal region there are large regions of the protein which exhibit very little identity to human or mouse CCL28. The bovine protein exhibited only ~23% identity to the mouse protein between amino acids 100–130 (Fig 1). In an effort to determine if bCCL28 exhibits antimicrobial activity, we generated recombinant protein for use in antimicrobial assays as previously described [11,13]. As a negative control, bCCL27 was used. CCL27 is closely related to CCL28 and like CCL28 mediates migration through binding the CCR10 chemokine receptor. However, unlike CCL28, CCL27 does not exhibit antimicrobial activity [11,12]. In *in vitro* killing assays both recombinant mCCL28 and bCCL28 functioned as effective antimicrobial proteins when tested against a common laboratory strain of *E*. *coli* (DH5α). Conversely, bCCL27 showed no antimicrobial activity (Fig 3).

**Fig 4:**
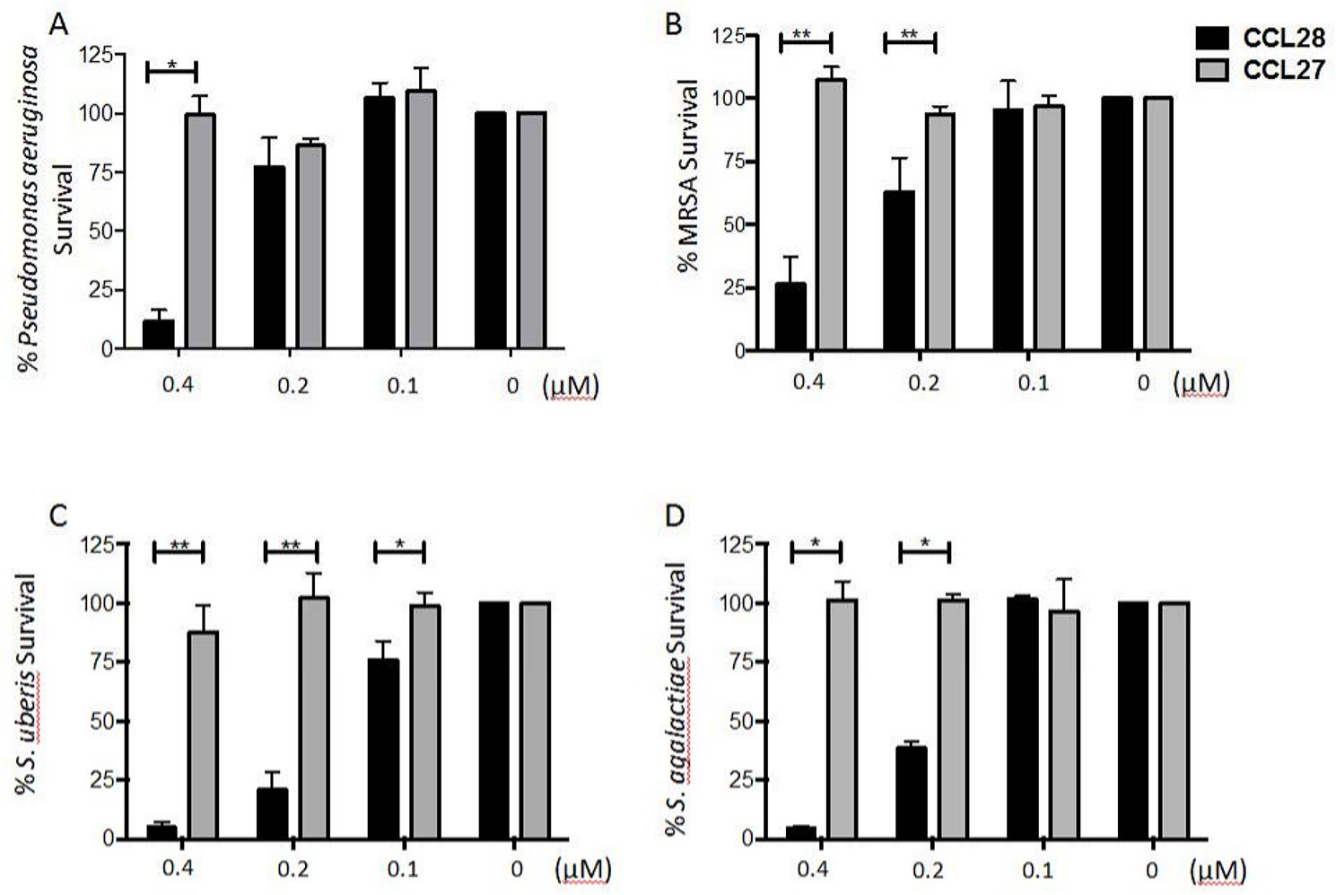
Recombinant bCCL28 has potent broad spectrum antimicrobial activity against mastitis causing bacteria. Bovine CCL28 demonstrates dose dependent antimicrobial activity against *Pseudomonas aeruginosa* (*A)*, methicillin-resistant *Staphylococcus aureus* (MRSA) *(B)*, *Streptococcus uberis (C)*, and *Streptococcus agalactiae (D)*. Bacteria were incubated with varied doses of bCCL28 or bCCL27 for 1 hour. Percent survival was calculated using the following equation: (CFU_pathogen + chemokine_/CFU_pathogen only_) x 100. *p<0.05, **p<0.01 as determined by Mann Whitney *U* test. Results are from four or more separate experiments. Error bars represent standard error of the mean. Details on percent survival, standard error, and the number of times each experiment was performed are available in S1 Fig.

Based on the effectiveness of bCCL28 in killing a non-clinical strain of *E*. *coli* we next tested the antimicrobial activity of bCCL28 on isolates of several bacterial strains commonly isolated from mastitic milk [26]. In these assays we analyzed the antimicrobial activity of bCCL28 against methicillin-resistant *Staphylococcus aureus* (MRSA), *Streptococcus uberis*, *Streptococcus agalactiae*, and *Pseudomonas aeruginosa*. We found that bCCL28 exhibited efficient killing of each species tested in a dose dependent manner (Fig 4A–4D). Consistent with the results seen using *E*. *coli*, bCCL27 demonstrated no antimicrobial activity.

### CCL28 levels in milk are highest soon after calving and do not correlate with somatic cell count

CCL28 has been found in human milk and is hypothesized to play a role as an antimicrobial protein in the protection of mucosal tissues [11]. In addition, our previous research has shown that bCCL28 mRNA is abundantly expressed in the bovine mammary gland [15]. To determine levels of bCCL28 protein produced *in vivo* we assessed CCL28 levels in bovine milk samples. Results from individual animals showed an average CCL28 concentration of 1.83 μg/ml (0.15 μM); the median CCL28 level in these samples was 1.43 μg/ml (0.11μM). CCL28 values from individual animals ranged from a low of 0.126 μg/ml (0.01 μM) to a high of 7.08 μg/ml (0.58 μM). Interestingly the levels of CCL28 measured in our assays are significantly higher than the CCL28 levels previously measured in human milk [11].

**Fig 5:**
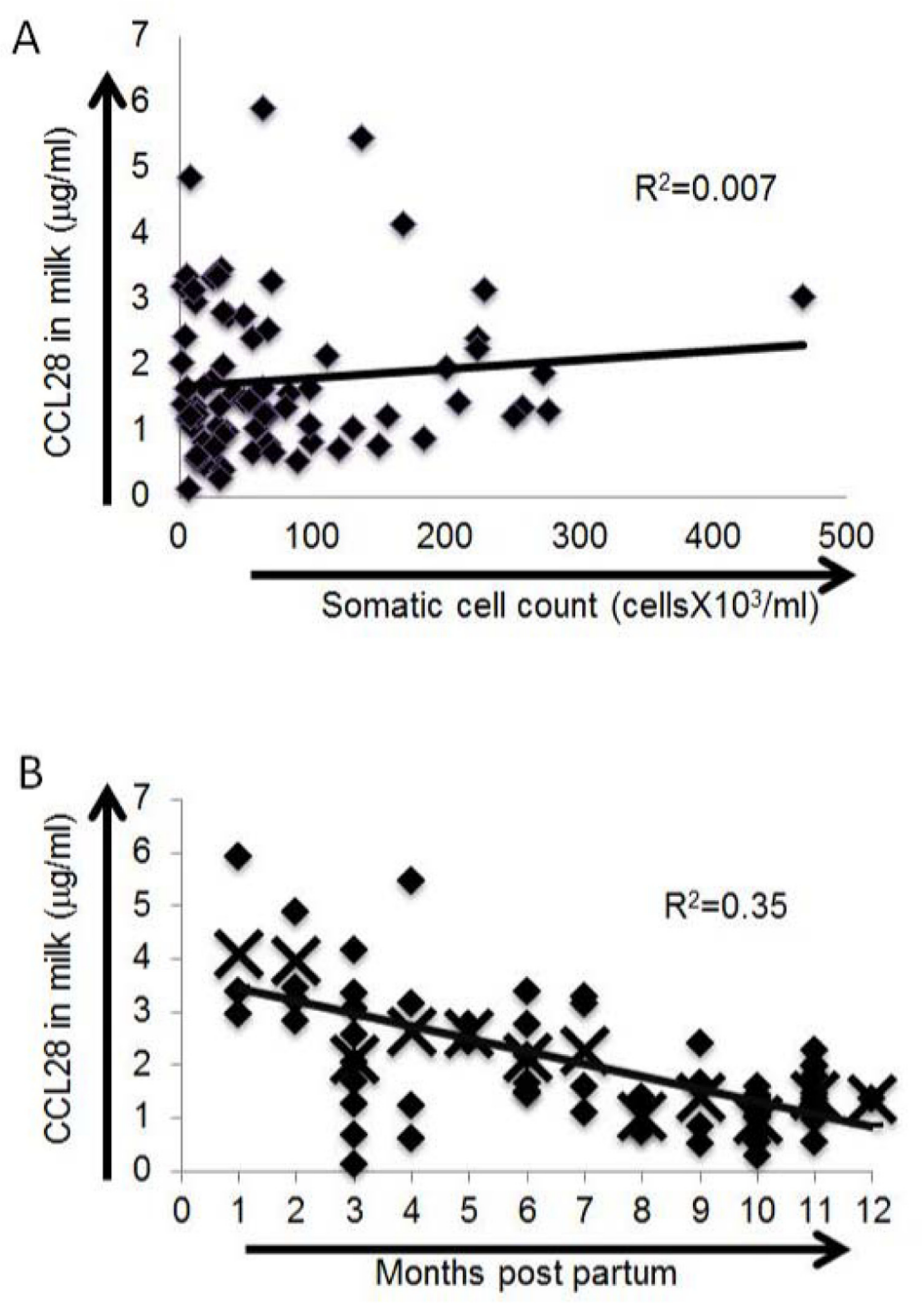
CCL28 levels in bovine milk are highest soon after parturition and are not correlated with somatic cell count. Milk CCL28 levels were determined by ELISA using antibodies generated against human CCL28. No correlation was seen between somatic cell counts and CCL28 protein levels in bovine milk (A). A strong correlation was seen (R^2^ = 0.35) between higher levels of CCL28 at the beginning of lactation (B). Diamonds represent individual milk samples. X’s represent the average CCL28 level for each time point indicated in the x-axis.

We tested for linear relationships between CCL28 levels in the milk and other measured indices. Somatic cell counts (high somatic cell counts are indicative of mastitis) were not linearly correlated with CCL28 levels (R^2^ = 0.007; Pearson’s correlation = 0.10, p = 0.39). Potentially, animals with somatic cell counts higher than those sampled in our study may exhibit an increase (or decrease) in milk CCL28 levels. No significant linear relationship was seen with cow age (Pearson’s correlation = 0.16, p = 0.20). Conversely, CCL28 levels were linearly weakly associated with pregnancy number (Pearson’s correlation = 0.28, p = 0.04) and very strongly associated with months postpartum (Pearson’s correlation = -0.59, p < 0.001). Transformation of the data did not increase linearity in months postpartum. Highest levels of CCL28 were seen early in lactation with protein levels decreasing an average of 0.24 μg/mL/month over time (R^2^ = 0.35, p < 0.001; Fig 5).

## Discussion

The study of chemokines has demonstrated that these molecules play vital roles in many aspects of immunity. These vital roles include cell survival, cell migration and accumulation, as well as functioning as antimicrobial proteins [27–29]. Most studies concerning the role of chemokines in immune function come from human and mouse models. Although mouse and human chemokines have been well characterized, the function of many bovine chemokines has yet to be experimentally validated [30,31]. Directly studying bovine chemokine function is essential to understanding the role of these indispensable regulators of immune function.

Previous research has shown that CCL28 mRNA is abundant in a variety of bovine tissues, including exocrine glands such as the mammary gland, parotid salivary gland and the mandibular salivary gland, as well as some intestinal tissues such as small and large intestine [15]. Although the chemokine is found in a variety of tissues, its principal receptor, CCR10, is predominantly found in a more restricted set of tissues. For example, although CCL28 is highly expressed in several bovine salivary glands, only the mandibular salivary gland expresses high levels of CCR10 [15]. The broad expression of CCL28 and more restricted expression of the CCR10 receptor has been interpreted by others to indicate that this chemokine likely serves a second function independent of its mediating the accumulation of lymphocytes via the CCR10 chemokine receptor [11].

The function of CCL28 as an antimicrobial peptide has been described previously [11,13,25]. Antimicrobial peptides are widespread in nature, occurring in organisms as diverse as plants and humans [14,32]. The C-terminal antimicrobial activity of CCL28 has been shown to be dependent on positively charged amino acid residues [13]. Structure function studies using mCCL28 suggest that although there are a number of positively charged amino acids in the C-terminal region, a particular series of five amino acids (RKDRK) is essential to the antimicrobial activity of the protein [13]. Data presented here demonstrates that although the bovine sequence varies at this region (RRNSK), the protein as a whole retains potent antimicrobial function. These results suggest that in this region, the charge of the amino acid is more important than the specific sequence. Additional studies are necessary to determine the exact properties required for the potent antimicrobial function of bCCL28.

Our investigation demonstrates bCCL28 has a strong antimicrobial effect against an array of pathogens which cause bovine mastitis. The susceptibility of these clinically relevant strains to bCCL28 indicates a potential role of this chemokine during infections of the lactating mammary gland. The direct killing of pathogens, coupled with the chemotactic properties of bCCL28, suggest a possible dual role for this chemokine in bovine immunity. Moreover, results of killing assays demonstrate that each of the pathogens tested were effectively killed at a concentration of 0.4μM CCL28. In many cases the levels of bCCL28 in bovine milk exceeded those required for significant antimicrobial activity *in vitro*, *s*uggesting CCL28 may contribute to mastitis prevention and/or recovery. Understanding the roles of specific cell types and proteins in preventing and/or recovering from mastitis is essential in designing and implementing mastitis prevention strategies.

Previous results have shown that mCCL28 is most effective as an antimicrobial protein in low salt solutions. Curiously, we do not see CCL28 mediated killing when assays are performed in whole milk (data not shown). We hypothesize the lactating mammary gland contains many microenvironments wherein the chemical milieu may vary dramatically. Potentially the CCL28 level may be very high on or near epithelial cells where the chemokine is produced. The chemical milieu (salt concentration) may also be more favorable to antimicrobial chemokine mediated killing in discrete microenvironments within the mammary gland.

Additional studies are needed to determine the exact role of CCL28 in bovine immunity. However, the lack of correlation between CCL28 and somatic cell counts suggests that mammary expression of CCL28 is likely not influenced by bacterial colonization. Alternatively, it is possible that infection with some, but not all, bacterial species stimulate an upregulation of CCL28 in the lactating mammary gland.

The extent of the antimicrobial role played by CCL28 in human, mouse, or cow immunity, *in vivo*, remains enigmatic. Here we demonstrate that bCCL28 is expressed in bovine milk at levels shown to kill mastitis-causing bacteria *in vitro*. Bovine CCL28 was also shown to mediate lymphocyte migration via the CCR10 chemokine receptor. This combined with the established role of mCCL28 in populating the mammary gland with antibody secreting cells suggests that bCCL28 plays a similar role in bovine mammary gland immunity. Furthermore, maternally produced CCL28 may provide passive immune protection to the nursing young and possibly play a role in shaping the bacterial colonization of the neonatal intestinal tract.

## Supporting Information

S1 Fig Statistical details for Fig 4. (TIF)
