# Supplementary figures and images for: Bovine CCL28 Mediates Chemotaxis via CCR10 and Demonstrates Direct Antimicrobial Activity against Mastitis Causing Bacteria
Source: PLoS One. 2015 Sep 11;10(9):e0138084. doi: 10.1371/journal.pone.0138084 (PMC4567263; doi:10.1371/journal.pone.0138084)

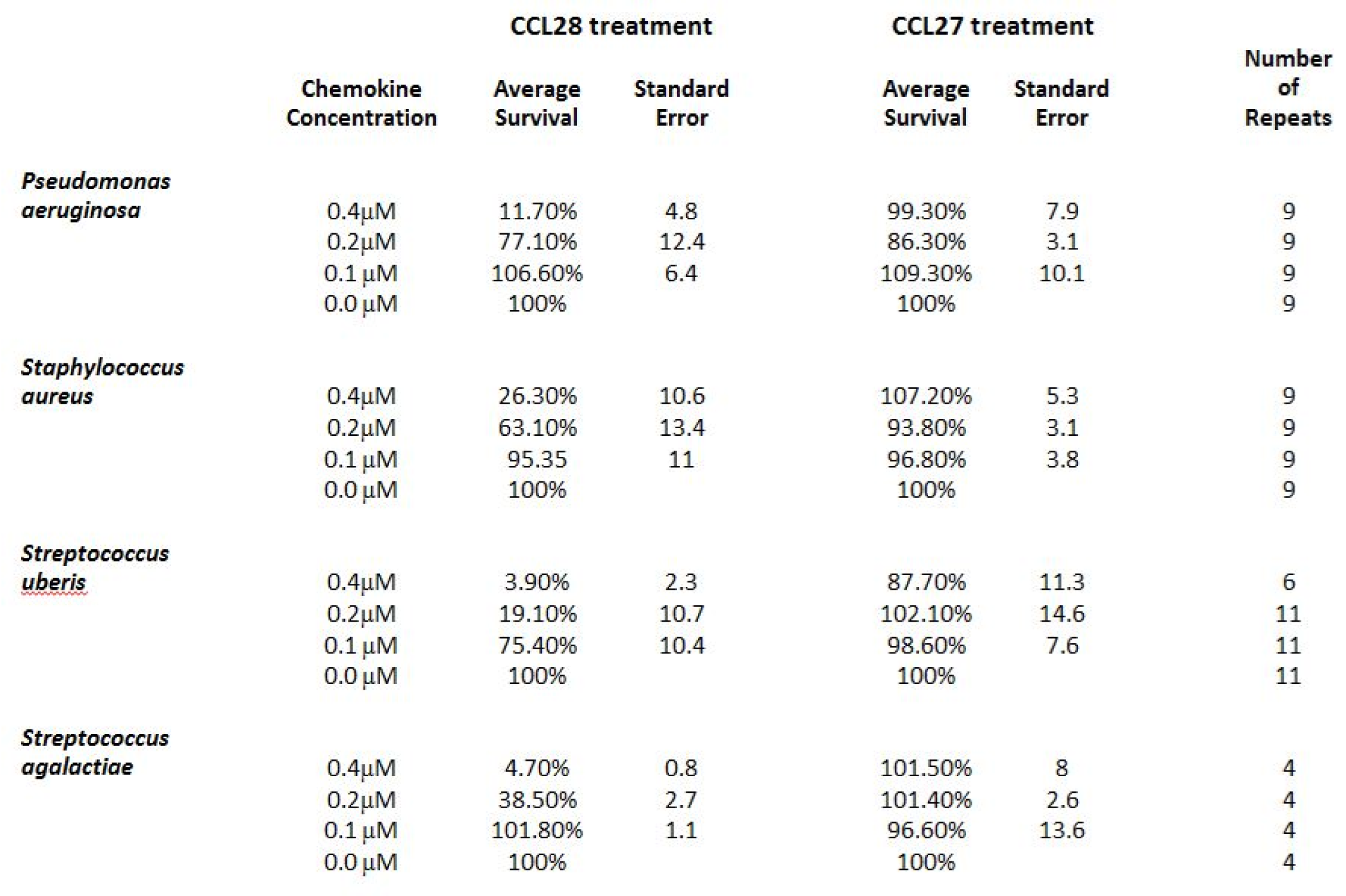

Supplement: S1 Fig — (TIF) [file pone.0138084.s001.tif]
